# Longitudinal Study on Post‐Traumatic Stress Disorder, Anxiety, and Depression Among Medical Staff in China

**DOI:** 10.1002/hcs2.70101

**Published:** 2026-09-13

**Authors:** Zongju Chen, Ruiting Cai, Yifang Liu, Na Tao, Yang Fei, Jing Wen, Pu Zhang, Yu Lu, Jun Zhang, Jing Wang, Li Zou, Wenning Fu

**Affiliations:** ^1^ School of Nursing, Tongji Medical College, Huazhong University of Science and Technology Wuhan China; ^2^ Department of Emergency Medicine Sichuan Academy of Medical Sciences & Sichuan Provincial People's Hospital Chengdu China; ^3^ Department of Social Medicine and Health Management School of Public Health, Tongji Medical College, Huazhong University of Science and Technology Wuhan China; ^4^ Department of Nursing The Central Hospital of Wuhan Wuhan China; ^5^ School of Nursing, Yangtze University Medical School Jingzhou China; ^6^ Department of Orthodontics West China Hospital of Stomatology, Sichuan University Sichuan China; ^7^ Department of Cardiology Taihe Hospital, Hubei University of Medicine Shiyan China; ^8^ Department of Endocrinology, Department of Cardiology Taihe Hospital, Hubei University of Medicine Shiyan China; ^9^ Reproductive Medical Center, Renmin Hospital of Wuhan University & Hubei Clinic Research Center for Assisted Reproductive Technology and Embryonic Development Wuhan China; ^10^ Department of Neurology Renmin Hospital of Wuhan University & Hubei Clinic Research Center for Assisted Reproductive Technology and Embryonic Development Wuhan China

**Keywords:** anxiety, cross‐lagged model, depression, medical staff, post‐traumatic stress disorder

## Abstract

**Background:**

Evidence of the longitudinal relationship between anxiety, depression, and post‐traumatic stress disorder (PTSD) is not uniform. This study aimed to explore the dynamic changes, independent factors, and bidirectional predictive relationships of anxiety, depression, and PTSD among medical staff.

**Methods:**

This study conducted a longitudinal follow‐up with two time points, 6 months apart, to collect medical staff's psychological data. Generalized linear mixed model and cross‐lagged model were used to identify independent factors of anxiety, depression, and PTSD among medical staff and explore longitudinal predictive relationships in main variables.

**Results:**

In total, 2650 medical staff completed both baseline and follow‐up surveys, with a follow‐up response rate of 96.3%. From the first phase of the survey (T1) to the second phase of the survey (T2), the prevalence of anxiety (28.9% at T1 and 34.5% at T2), depression (19.4% and 26.7%), and PTSD (6.1% and 13.1%) among medical staff showed an upward trend. Occupational exposure, sleep quality, and experiencing traumatic life events were identified as independent factors associated with psychological disorders among medical staff. In addition, from T1 to T2, PTSD predicted anxiety and depression, and depression predicted PTSD and anxiety. However, anxiety could not predict depression and PTSD.

**Conclusions:**

The prevalence of anxiety, depression, and PTSD among medical staff was higher and showed an upward trend. It is recommended to conduct early screening and intervention for medical staff's mental health problems, while paying close attention to the comprehensive management and intervention of these symptoms.

## Graphical abstract

A six‐month study of 2650 medical staff found post‐traumatic stress disorder and depression bidirectionally predicted each other, and both drove later anxiety. These findings highlight the need for integrated early screening and intervention strategies targeting post‐traumatic stress disorder, anxiety and depression in high‐stress healthcare settings.

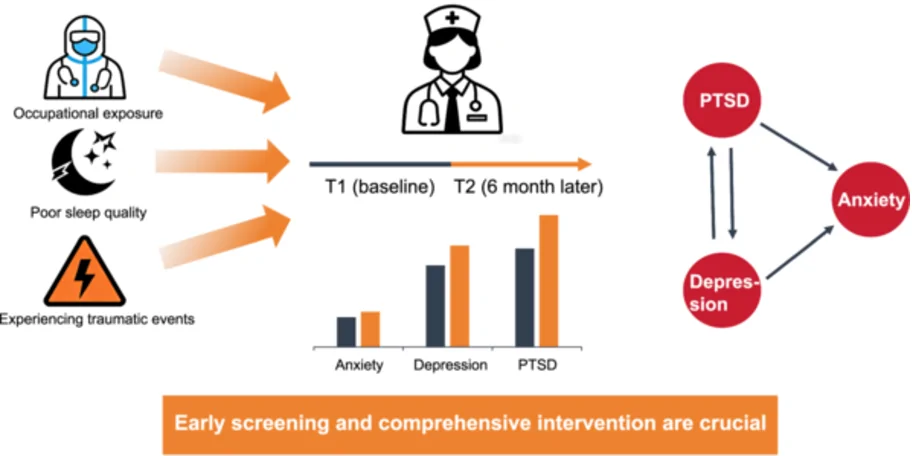

**Abbreviations**
AOR: adjusted odds ratio
CES‐D: Center for Epidemiologic Studies Depression Scale
CI: confidence interval
DSM‐5: Diagnostic and Statistical Manual of Mental Disorders, Fifth Edition
GAD‐7: generalized anxiety disorder‐7
GLMM: generalized linear mixed model
PCL‐5: PTSD Checklist for DSM‐5
PTSD: post‐traumatic stress disorder
SD: standard deviation
T1: the first phase of the survey
T2: the second phase of the survey

## 1 Background

Medical staff suffered from various stressful events during clinical work, such as heavy workloads, tense physician‐patient relationships and nurse–patient relationships, workplace violence, and prolonged feelings of distress arising from constant exposure to mortality [1, 2, 3], which continually impose heavy burdens on their mental and psychological health, resulting in a persistent state of high tension and stress. Furthermore, these occupational specificities may cause the dynamic relationships among the mental health problems to differ from those observed in the general population or other occupational groups. In China, these occupational stressors may be particularly salient due to several contextual factors. In particular, Chinese medical staff face unique barriers to seeking mental health support. Previous research showed that under significant psychological pressure, medical staff in China tend to have more negative attitudes toward professional psychological help‐seeking, with mental illness stigma and fear of discrimination from colleagues or patients being key contributing factors [4, 5]. These cultural and systemic characteristics distinguish the Chinese healthcare context from that of Western countries and highlight the necessity of examining the mental health of medical staff specifically within the Chinese setting.

Previous studies revealed that, due to various stressful events, medical staff often suffer from immediate and long‐term mental and psychological consequences like post‐traumatic stress disorder (PTSD), anxiety, and depression [6, 7, 8, 9], and even suicidal behaviors [10], thus greatly increasing the burden on the healthcare system. A systematic review focusing specifically on Chinese medical staff reported a substantial prevalence of psychological issues among medical staff, with aggregate prevalence rates of anxiety symptoms (27.0%) and depression symptoms (26.2%) [11]. Another meta‐analysis found that the overall prevalence of PTSD among Chinese medical staff during the pandemic was 29.2% [12]. These figures highlight the urgent need for comprehensive mental health surveillance and intervention among Chinese medical staff. However, the majority of existing studies that focused on the mental and psychological disorders among medical staff employed single‐center cross‐sectional research designs [13]. These studies failed to provide dynamic changes in the mental and psychological status of medical staff over time and were unable to directly determine the predictive relationships between variables. Therefore, conducting long‐term monitoring of mental and psychological consequences, including anxiety, depression, and PTSD symptoms, among medical staff for better early assessment and intervention is essential.

In addition, the longitudinal predictive relationships between anxiety, depression, and PTSD symptoms were complex and controversial. Previous studies found that PTSD symptoms have a unidirectional predictive relationship with depression [14, 15]. In contrast, some scholars believe that depression and PTSD symptoms may be mutually causal [16]. Furthermore, previous research has suggested that anxiety symptoms precede depression symptoms [17]. However, some scholars have argued that the relationship between anxiety and depression symptoms is not unidirectional, but rather bidirectional [18]. Identifying and understanding the longitudinal predictive relationships between anxiety, depression, and PTSD symptoms among medical staff facilitates comprehensive management and intervention by administrators in addressing their mental health status.

Considering the deficiency of existing research on the predictive relationships of mental and psychological disorders in medical staff, this study adopted a longitudinal research design to follow up on the anxiety, depression, and PTSD symptoms in medical staff from two general hospitals in Hubei Province, China, for a period of 6 months. Our study aimed to confirm the dynamic changes of anxiety, depression, and PTSD among medical staff, identify risk factors that were independently associated with anxiety, depression, and PTSD, and explore the longitudinal predictive relationships between anxiety, depression, and PTSD symptoms, thus providing scientific reference bases with which to develop and optimize precise psychological intervention strategies for medical staff.

## 2 Methods

### 2.1 Design and Participants

The current research was conducted utilizing an online survey tool, namely “Survey Star” (Changsha Ranxing Science and Technology, Changsha, China). To guarantee the accuracy and usability of the collected data, we incorporated an intelligent logical validation process within the computer back‐end system, aimed at identifying and excluding invalid questionnaires. Each participant was restricted to completing the survey only once, thereby ensuring the uniqueness and quality of the data.

A longitudinal follow‐up research design was utilized to survey the mental and psychological status of medical staff. First, two tertiary‐grade A general hospitals in Hubei Province, China—one a regional medical center and the other a prefecture‐level municipal hospital—were selected using convenience sampling. Subsequently, online questionnaires were distributed to healthcare workers through the medical and nursing departments. The inclusion criteria for this study were age ≥ 18 years, possession of the required practicing certificate, and voluntary project participation. Participants with PTSD, anxiety disorders, depression, and so on, who were diagnosed within 6 months by a general hospital of the second level or above or a hospital specializing in psychiatry, according to the International Classification of Diseases 11th Revision [19], were excluded. Also excluded were those who changed their work locations within 6 months of the start of the project.

The first phase of the survey (T1) was conducted in May 2022, with 2751 medical staff members completing the questionnaires. All participants were informed that they might be contacted again after 6 months for the second round of assessment. The same medical staff were invited to complete the same questionnaire 6 months later (the second phase of the survey [T2]). After excluding the questionnaires of 101 participants who either requested withdrawal from follow‐up, were lost to follow‐up upon resignation, showed evidence of duplication, or gave invalid responses, 2650 valid questionnaires were collected, with a follow‐up response rate of 96.3%.

The present study adhered to the principles of the Declaration of Helsinki, as revised in 2013. All the participants were asked to read and sign an informed consent form before accessing the link to complete the questionnaire. This study was approved by the Research Ethics Committee of the Tongji Medical College, Huazhong University of Science and Technology, Wuhan, China (No. 2021‐IEC‐A006). The ethical approval from Tongji Medical College, Huazhong University of Science and Technology has been acknowledged by the other participating hospitals.

### 2.2 Measurements

#### 2.2.1 Sociodemographic Information

A questionnaire focusing on general demographic characteristics was formulated based on an extensive literature review. The primary variables were age, sex, level of education, occupation, marital status, department, occupational exposure, sleep quality, exercise, and so on.

#### 2.2.2 The PTSD Checklist for Diagnostic and Statistical Manual of Mental Disorders, Fifth Edition (DSM‐5) (PCL‐5)

In this study, PTSD was defined as a persistent and maladaptive psychological condition that occurs after medical staff experience or are repeatedly exposed to potentially life‐threatening, severely physically or psychologically traumatic, or profoundly helplessness‐inducing work‐related events in clinical settings (e.g., facing patient death, exposure to workplace violence, and prolonged engagement in high‐stress doctor–patient or nurse–patient environments). PTSD was assessed using the PCL‐5. This scale was developed by Blevins et al. based on the diagnostic criteria for PTSD in the DSM‐5, with an internal consistency of 0.94 and a test–retest reliability of 0.82 [20]. The PCL‐5 comprises 20 items, categorized into four symptom clusters: re‐experiencing, avoidance, negative alterations in cognition or mood, and hyperarousal. The PCL‐5 responses are made on a 5‐point Likert scale where each item is rated from 0 (*never*) to 4 (*extremely*). A cut‐off score of ≥ 33 indicates probable PTSD diagnosis. An elevated PTSD score signifies increased severity of PTSD symptoms. In the present study, the Cronbach's alpha values for the PCL‐5 questionnaire were 0.982 for T1 and 0.989 for T2.

#### 2.2.3 Generalized Anxiety Disorder‐7 (GAD‐7)

This questionnaire was developed by Spitzer et al. based on the Diagnostic and Statistical Manual of Mental Disorders, Fourth Edition to evaluate the severity of individual anxiety symptoms, with an internal consistency of 0.92 and a test–retest reliability of 0.83 [21]. The GAD‐7 comprises seven items, with a scoring range from 0 to 21, utilizing a four‐point Likert scale where each item is rated from 0 (*not at all*) to 3 (*nearly every day*). Higher scores indicate more severe anxiety symptoms. In this study, a score of 5 was used as the cut‐off score for positive anxiety symptoms. In our study, the Cronbach's alpha values for the GAD‐7 scale were 0.955 for T1 and 0.971 for T2.

#### 2.2.4 Center for Epidemiologic Studies Depression Scale (CES‐D)

The CES‐D questionnaire was developed by Radloff, with an internal consistency of 0.85 and a test–retest reliability of 0.67 [22]. It is primarily used to screen depression symptoms among the general population. This scale consists of four dimensions: depression emotions, physical symptoms, positive emotions, and interpersonal relationships. Comprising 20 items, the CES‐D scale offers a total score of 0–60, utilizing a 4‐point Likert scale where scores range from 0 (*basically not*, < 1 day/week) to 3 (*always*, 5–7 days/week) for each item. Items 4, 8, 12, and 16 were reverse‐scored. A cutoff score of 16 was employed to indicate the presence of positive depression symptoms. In the current research, the Cronbach's alpha values for this CES‐D questionnaire were 0.978 for T1 and 0.984 for T2.

### 2.3 Statistical Analysis

SPSS 23.0 (IBM Corporation, Armonk, United States) and Stata MP 17 (StataCorp LLC, College Station, United States) were used for the statistical analysis. Prior to proceeding, we verified the normality of all variables according to the criteria established by Curran et al. [23]. Our findings confirmed that all variables in this study adhered to the assumption of normal distribution. Continuous variables are described as mean ± standard deviation (SD), and categorical variables are described as frequencies (*n*) and percentages (%). A chi‐square test was adopted to compare the demographic characteristics of the participants who completed the surveys with those who did not complete them, aiming to analyze structural losses in the survey sample, as well as to identify factors independently associated with anxiety, depression, and PTSD symptoms. Pearson's correlation analysis was employed to examine the associations between anxiety, depression, and PTSD symptoms, both cross‐sectionally and longitudinally. Statistical significance was set at *p* < 0.05 (two‐tailed).

Given the longitudinal design with repeated measures at baseline and follow‐up, we employed generalized linear mixed models (GLMMs) with a logit link to identify independent risk factors for anxiety, depression, and PTSD symptoms. To correctly handle the repeated measures within each participant, a random intercept for each medical staff member was included in all models. This accounts for the within‐individual correlation across the two time points. Time (baseline vs. follow‐up) was treated as a fixed effect to estimate the overall change in outcomes across the study period. Recognizing that medical staff were nested within different hospitals, which might introduce cluster‐level effects, we initially included a random intercept for hospital in the model. This approach allows for the estimation of between‐hospital variance while efficiently handling the multi‐center data structure. All models successfully converged. Variables showing association (*p* < 0.05) in preliminary univariate analyses, along with those deemed clinically relevant, were offered as candidate independent variables. The final multivariable GLMMs were constructed to evaluate independent risk factors, presented as adjusted odds ratios (AORs) with their 95% confidence intervals. A total of 2650 medical staff members who completed both the baseline and follow‐up surveys were included in the analysis.

Mplus 8.3 (Muthén & Muthén, Los Angeles, United States) was used to construct a cross‐lagged model to explore the bidirectional predictive relationships between anxiety, depression, and PTSD symptoms from T1 to T2. The cross‐lagged model is based on the design of a cross‐lagged panel and is a statistical analysis method that explores the correlation between variables in a time series based on longitudinal tracking data using a robust maximum likelihood iteration procedure. This method can more accurately infer causal relationships that are closer to the truth. Determination of model fit followed standard recommendations according to the following fit indices [24]: the *χ* ^2^/df index < 3, Comparative Fit Index ≥ 0.90, Tucker–Lewis Index ≥ 0.90, root‐mean‐square error of approximation ≤ 0.08, and standardized root mean square residual ≤ 0.08.

## 3 Results

In the present study, a total of 2650 medical staff completed the longitudinal follow‐up, with a follow‐up response rate of 96.3%. Among the 2650 medical staff members who completed the longitudinal follow‐up, 83.2% (2206) of medical staff were female, approximately half (51.7%) were aged 31–40 years, 80.3% (2128) were nurses, and 13.1% (348) of medical staff reported having experienced occupational exposure in their clinical work during the previous year. Table 1 presents the participants' demographic characteristics and the results of the chi‐square test.

**Table 1:** Demographic characteristics of the participants (*n* = 2650).

		*χ* ^2^		
Variables	*n* (%)	Anxiety	Depression	PTSD
Sex		1.125	10.357^b^	26.000^c^
Male	444 (16.8)			
Female	2206 (83.2)			
Age (years)		11.363^b^	3.669	3.032
20–30	890 (33.6)			
31–40	1370 (51.7)			
> 40	390 (14.7)			
Marital status		3.925	0.674	2.051
Single	639 (24.1)			
Married	1964 (74.1)			
Others	47 (1.8)			
Level of education		1.042	10.371^b^	8.003^a^
Below bachelor's degree	150 (5.6)			
Bachelor's degree	1963 (74.1)			
Master's degree and above	537 (20.3)			
Type of occupation		1.549	10.149^b^	15.440^c^
Doctor	504 (19.0)			
Nurse/midwife	2128 (80.3)			
Others	18 (0.7)			
Department		9.080^a^	2.903	3.674
Emergency	69 (2.6)			
Intensive care unit	182 (6.9)			
Others	2399 (90.5)			
Average monthly income (CNY)		5.196	5.924	1.045
< 5000	167 (6.3)			
5000–9999	1284 (48.5)			
10,000–14,999	890 (33.6)			
≥ 15,000	309 (11.6)			
Occupational exposure		17.763^c^	21.927^c^	20.561^c^
Yes	348 (13.1)			
No	2302 (86.9)			
Exercise		5.255^a^	9.610^b^	0.305
Always	613 (23.1)			
Little or no	2037 (76.9)			
Sleep quality		117.610^c^	99.601^c^	47.441^c^
Good	623 (23.5)			
General	1623 (61.2)			
Poor	346 (13.1)			
Very poor	58 (2.2)			
Experiencing traumatic life events		34.825^c^	27.429^c^	26.338^c^
Yes	363 (13.7)			
No	2287 (86.3)			

*Note:* Data are presented as *n* (%).

Abbreviations: CNY, Chinese Yuan; PTSD, post‐traumatic stress disorder.

^a^ *p* < 0.05.

^b^ *p* < 0.01.

^c^ *p* < 0.001.

Table S1 shows the demographic characteristics of the medical staff who completed the follow‐up, as well as those who did not. In this study, the sample losses were random, without structural losses, and they did not significantly affect the data analysis results.

From T1 to T2, the symptom scores (anxiety, 2.90 ± 3.80 at T1 and 3.43 ± 4.42 at T2; depression, 7.97 ± 12.09 at T1 and 9.92 ± 13.79 at T2; PTSD, 9.63 ± 12.77 at T1 and 12.87 ± 16.25 at T2) and the prevalence (anxiety, 28.9% at T1 and 34.5% at T2; depression, 19.4% at T1 and 26.7% at T2; PTSD, 6.1% at T1 and 13.1% at T2) of anxiety, depression, and PTSD symptoms among medical staff showed an upward trend (Figures 1 and 2).

**Figure 1:**
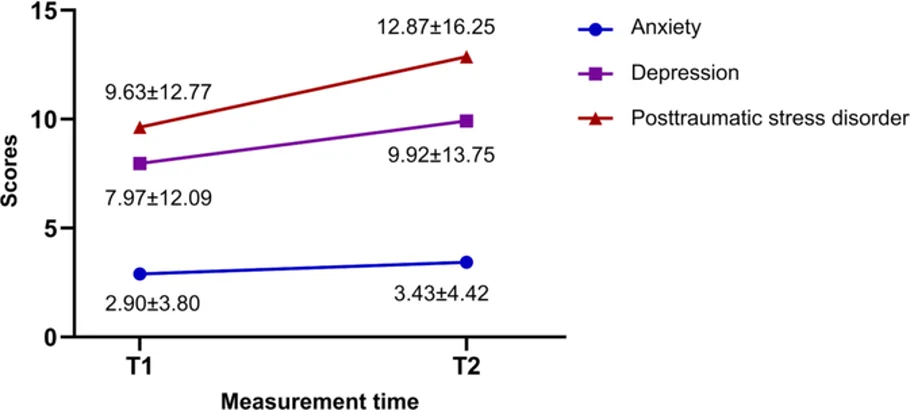
The scores of anxiety, depression, and PTSD symptoms at T1 and T2 among medical staff (*n* = 2650). PTSD, post‐traumatic stress disorder; T1, the first phase of the survey; T2, the second phase of the survey.

**Figure 2:**
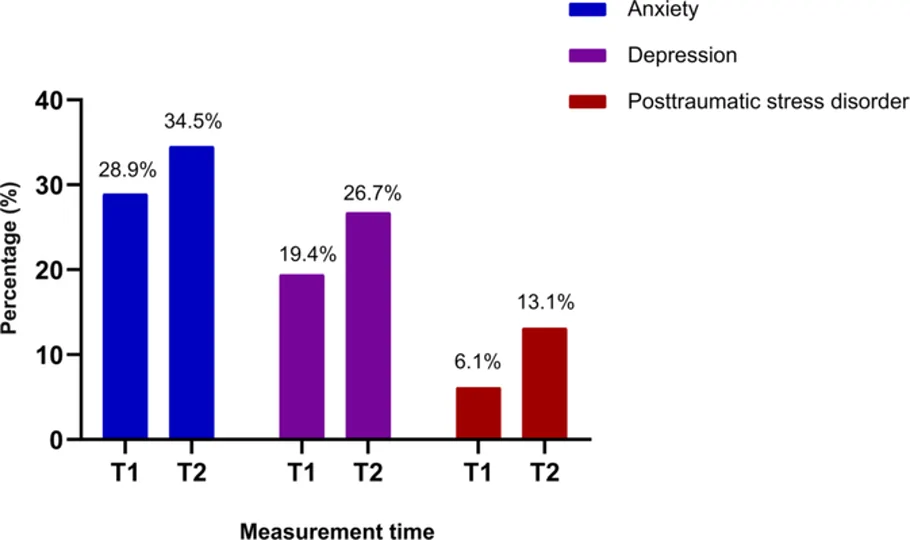
The positive detection rates of anxiety, depression, and PTSD symptoms at T1 and T2 among medical staff (*n* = 2,650). PTSD, post‐traumatic stress disorder; T1, the first phase of the survey; T2, the second phase of the survey.

Subsequently, a GLMM was used to identify the independent risk factors of anxiety, depression, and PTSD symptoms among medical staff. The GLMM results are presented in Table 2. According to the GLMM analysis, risk factors that were independently associated with anxiety included age, occupational exposure, sleep quality, and experiencing traumatic life events. Specifically, medical staff aged 31–40 years (AOR = 1.26, 95% confidence interval [CI]: 1.10–1.44) or aged > 40 years (AOR = 1.37, 95% CI: 1.13–1.65), experiencing occupational exposure (AOR = 1.51, 95% CI: 1.27–1.79), experiencing traumatic life events (AOR = 1.71, 95% CI: 1.45–2.02), and those who had general, poor, or very poor sleep quality (AOR = 2.14, 95% CI: 1.82–2.53; AOR = 3.75, 95% CI: 3.02–4.65; AOR = 7.82, 95% CI: 5.16–11.86, respectively) had a higher risk of anxiety disorders. Risk factors that were independently associated with depression included occupational exposure, sleep quality, and experiencing traumatic life events. Specifically speaking, medical staff experiencing occupational exposure (AOR = 1.47, 95% CI: 1.22–1.76), experiencing traumatic life events (AOR = 1.66, 95% CI: 1.39–1.98), and those who had general (AOR = 1.79, 95% CI: 1.49–2.15), poor (AOR = 3.08, 95% CI: 2.44–3.88), or very poor sleep quality (AOR = 7.66, 95% CI: 5.09–11.53) had a higher risk of depression. Sex, occupational exposure, sleep quality, and experiencing traumatic life events were identified as independent factors associated with PTSD among medical staff. More specifically, male (AOR = 0.62, 95% CI: 0.47–0.83) medical staff were less likely to develop PTSD symptoms, whereas medical staff experiencing occupational exposure (AOR = 1.50, 95% CI: 1.18–1.92), experiencing traumatic life events (AOR = 1.63, 95% CI: 1.28–2.06), and those who had general (AOR = 1.36, 95% CI: 1.06–1.75), poor (AOR = 1.97, 95% CI: 1.44–2.70), or very poor sleep quality (AOR = 2.32, 95% CI: 1.32–4.08) had a higher risk of PTSD symptoms.

**Table 2:** The results of generalized linear mixed model (*n* = 2650).

	Anxiety		Depression		PTSD	
Variables	AOR (95% CI)	*p*	AOR (95% CI)	*p*	AOR (95% CI)	*p*
Sex						
Female	—	—	Ref	—	Ref	—
Male	—	—	0.84 (0.67–1.04)	0.113	0.62 (0.47–0.83)	0.001
Age (years)						
20–30	Ref	—	—	—	—	—
31–40	1.26 (1.10–1.44)	0.001	—	—	—	—
> 40	1.37 (1.13–1.65)	0.001	—	—	—	—
Level of education						
Below bachelor's degree	—	—	Ref	—	Ref	—
Bachelor's degree	—	—	1.37 (0.99–1.87)	0.053	1.17 (0.76–1.82)	0.476
Master's degree and above	—	—	1.31 (0.86–2.00)	0.208	1.02 (0.57–1.84)	0.945
Type of occupation						
Doctor	—	—	Ref	—	Ref	—
Nurse/midwife	—	—	0.86 (0.62–1.19)	0.363	0.75 (0.48–1.17)	0.206
Others	—	—	0.42 (0.16–1.14)	0.089	0.17 (0.02–1.29)	0.087
Department						
Emergency	Ref	—	—	—	—	—
Intensive care unit	1.52 (0.99–2.37)	0.057	—	—	—	—
Others	1.24 (0.84–1.81)	0.280	—	—	—	—
Occupational exposure						
No	Ref	—	Ref	—	Ref	—
Yes	1.51 (1.27–1.79)	< 0.001	1.47 (1.22–1.76)	< 0.001	1.50 (1.18–1.92)	0.001
Exercise						
Little or no	Ref	—	Ref	—	—	—
Always	0.87 (0.74–1.01)	0.062	0.87 (0.73–1.03)	0.095		
Sleep quality						
Good	Ref	—	Ref	—	Ref	—
General	2.14 (1.82–2.53)	< 0.001	1.79 (1.49–2.15)	< 0.001	1.36 (1.06–1.75)	0.014
Poor	3.75 (3.02–4.65)	< 0.001	3.08 (2.44–3.88)	< 0.001	1.97 (1.44–2.70)	< 0.001
Very poor	7.82 (5.16–11.86)	< 0.001	7.66 (5.09–11.53)	< 0.001	2.32 (1.32–4.08)	0.003
Experiencing traumatic life events						
No	Ref	—	Ref	—	Ref	—
Yes	1.71 (1.45–2.02)	< 0.001	1.66 (1.39–1.98)	< 0.001	1.63 (1.28–2.06)	< 0.001

Abbreviations: AOR, adjusted odds ratio; CI, confidence interval; PTSD, post‐traumatic stress disorder; Ref, reference; —, not applicable.

Table S2 shows the correlations between anxiety, depression, and PTSD symptoms at T1 and T2. The anxiety, depression, and PTSD symptoms all showed a significant positive correlation at T1 and T2 (*p* < 0.01).

Figure 3 presents the results of the cross‐lagged analysis of anxiety, depression, and PTSD symptoms. Owing to the saturated model, the estimation of all parameters coincided with the elements of the covariance matrix, resulting in zero degrees of freedom. Therefore, only path coefficients were considered, and the model fit evaluation metrics were not estimated [25]. The autoregressive coefficients of anxiety, depression, and PTSD symptoms were significant at T1 and T2 (*p* < 0.001), with values of 0.414, 0.157, and 0.232, respectively. From T1 to T2, PTSD symptoms predicted anxiety and depression symptoms (*β* = 0.289, 0.221; *p* < 0.001), and depression symptoms predicted PTSD and anxiety symptoms (*β* = 0.084, 0.079; *p* < 0.001). However, anxiety symptoms could not predict depression and PTSD symptoms (*β* = 0.017, 0.039; *p* > 0.05). In addition, a Wald test was conducted to compare the magnitudes of the two cross‐lagged paths between depression and PTSD. The results showed that the path from PTSD to depression (*β* = 0.221) was significantly stronger than the reverse path from depression to PTSD (*β* = 0.084), with a difference of −0.126 (Wald *χ* ^2^ = 6.36, *p* = 0.012).

**Figure 3:**
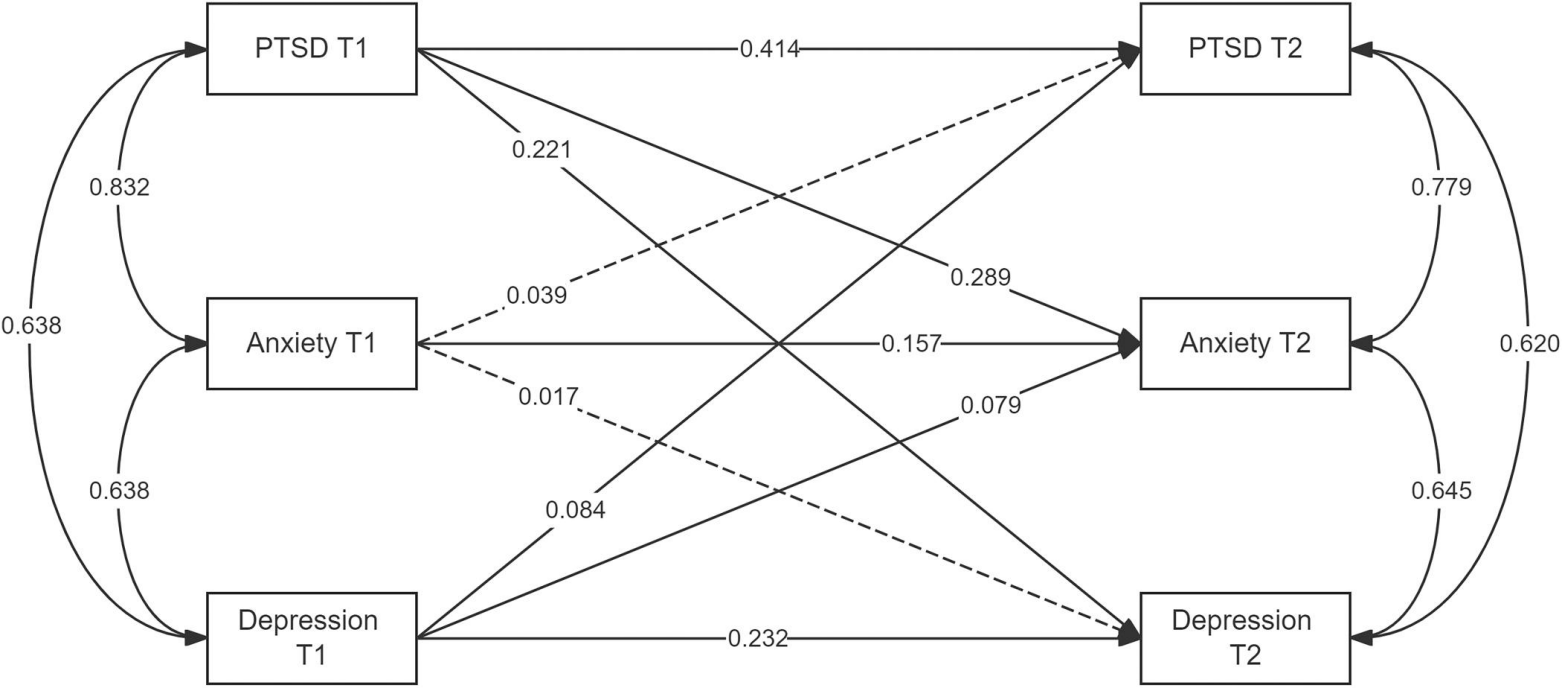
Cross‐lagged model of associations between PTSD, anxiety and depression symptoms. The data next to the connecting line are standardized regression coefficients (*β*). The solid line represents statistically significant regression coefficients (*p* < 0.05), while the dashed line represents non‐significant regression coefficients (*p* > 0.05). PTSD, post‐traumatic stress disorder; T1, the first phase of the survey; T2, the second phase of the survey.

## 4 Discussion

In this study, we conducted a longitudinal follow‐up to explore the mental and psychological disorders of medical staff coming from two hospitals in Hubei Province, China. We found that the prevalence of anxiety, depression, and PTSD symptoms among medical staff is higher and increasing. In addition, there were bidirectional predictive relationships between PTSD and depression symptoms among medical staff, while PTSD/depression symptoms only unidirectionally predicted anxiety disorders. This study advances the understanding of anxiety, depression, and PTSD symptoms among medical staff and highlights evidence explaining the bidirectional predictive relationships between anxiety, depression, and PTSD symptoms.

In this study, we found that the levels and prevalence of anxiety, depression, and PTSD symptoms among medical staff show an upward trend during the follow‐up period, indicating a deteriorating mental health status. Heavy workload, tense doctor–patient relationships, and so on can exacerbate the mental and psychological disorders of medical staff [1, 2]. Notably, the follow‐up period of this study coincided with the COVID‐19 pandemic in Hubei Province, during which several regions were adjusted from low‐risk to medium‐risk or high‐risk status. This shift in risk level may have imposed additional stress on healthcare workers, contributing to the observed fluctuations in their mental health. Previous research has also supported this notion, indicating that medical staff's negative emotions tend to fluctuate over the duration of a pandemic [26, 27, 28]. Furthermore, the negative impact of major traumatic events on mental health will persist for a long time [6, 27]. Thus, long‐term mental health monitoring is important. It is necessary to classify medical staff with high levels of anxiety, depression, and PTSD symptoms as key populations and to conduct continuous mental health monitoring, and promptly provide early prevention and intervention measures to improve medical staff's mental health, thereby enhancing their work efficiency and quality.

According to the results of GLMM analysis, sleep quality, experiencing occupational exposure, and experiencing major traumatic life events were identified as independent factors associated with anxiety, depression, and PTSD symptoms among medical staff. Walker argued that sleep performs a function similar to systematic desensitization therapy [29]. In other words, sleep normally serves to remove the emotional component from memories. A failure or an absence of this process may trigger mental and psychological symptoms. A systematic review and meta‐analysis by Alimoradi et al., which included 345,270 participants from 39 countries, reported that sleep problems were associated with depression and anxiety among healthcare professionals. Of note, the same study also found that sleep problems were associated with higher levels of psychological distress and suggested that treating sleep problems may reduce psychological distress, and vice versa, pointing to a bidirectional relationship between sleep and mental health [30]. Future research should adopt longitudinal designs with multiple time points to clarify the directionality between sleep problems and mental health symptoms among medical staff, and to examine whether interventions targeting sleep can effectively reduce subsequent psychological distress.

Besides, this study found that occupational exposure positively predicted mental health problems among medical staff. Occupational exposure among healthcare workers is not only pandemic‐related exposure but also workplace violence, needlestick injuries, exposure to patient death and suffering, and moral injury. A rapid review and meta‐analysis by Bell et al., which included 74 studies from outbreaks of COVID‐19, Ebola, Middle East Respiratory Syndrome and Severe Acute Respiratory Syndrome, reported that clinical staff working in high‐exposure roles had slightly higher levels of acute mental health problems than those in low‐exposure roles [31]. A systematic review by Liu et al. examining workplace violence against healthcare professionals concluded that workplace violence might lead to various negative impacts on health workers' psychological health, such as increases in stress and anxiety levels and burnout [32]. Hospital administrators should pay more attention to the effect of occupational exposure on medical staff's physical and mental health. Targeted support, such as regular psychological debriefing for staff in high‐exposure roles and accessible reporting systems for workplace violence, could help mitigate these negative outcomes.

This study also found that experiencing major traumatic life events within the past year, such as serious illness or death of a family member, significantly predicted elevated levels of anxiety, depression, and PTSD among medical staff. Medical staff are routinely exposed to ongoing occupational stressors, including high workload, tense doctor–patient relationships, and exposure to patient suffering and death, which may weaken their psychological resilience over time. When a major family‐related traumatic event occurs on top of this occupational context, the additive or interactive effect may produce an increase in anxiety, depression, and PTSD symptoms relative to either type of stress alone. Notably, the existing literature has primarily focused on work‐related trauma among medical staff, including critical incidents in hospitals and large‐scale events such as pandemics and natural disasters. However, major traumatic events occurring in the personal domain, such as serious illness or death of a family member, have received less research attention. One possible reason for this gap is that these personal events have been implicitly treated as non‐occupational and thus excluded from workplace mental health research. Therefore, managers should recognize and acknowledge the impact of major traumatic life events on medical staff's mental health, and actively encourage staff to voluntarily report such events and seek timely support to alleviate psychological distress.

The bidirectional predictive relationships between anxiety, depression, and PTSD among medical staff were evaluated in our study. In line with previous research [33, 34], we found a bidirectional predictive relationship between depression and PTSD symptoms among medical staff from T1 to T2. Beyond simply confirming the bidirectional association, a comparison of the two cross‐lagged paths revealed notable differences in their magnitudes: the path from PTSD to depression was approximately three times stronger than the reverse path. This result is consistent with causal discovery analyses reported by Pierce et al., who found consistent, directed associations from PTSD symptoms to depressive symptoms across five longitudinal cohorts [35]. These findings suggest that PTSD symptoms may exert a stronger temporal influence on depression than vice versa. One possible reason is that PTSD‐related hyperarousal, avoidance, and negative alterations in cognition and mood directly impair daily functioning and reduce engagement in activities, thereby increasing the risk of depression [35]. However, using a three‐wave longitudinal design following Chinese adolescents after the Yancheng tornado, An et al. found that PTSD symptoms significantly predicted subsequent depression symptoms, whereas depression did not significantly predict PTSD [36]. Another study employing network analysis found that PTSD and depression are two separate disorders [37]. The heterogeneity across studies may be due to differences in sample characteristics, trauma types, and follow‐up intervals. Therefore, future longitudinal studies with three or more waves are needed to replicate our findings and to test whether the asymmetric pattern holds across different populations.

Studies of the temporal correlation between PTSD and anxiety symptoms are limited. One study found that PTSD symptoms in migrant populations significantly predicted subsequent anxiety symptoms during a pandemic; however, anxiety symptoms could not predict subsequent PTSD symptoms [38]. This study further supports the conclusion that PTSD unidirectionally predicts subsequent anxiety symptoms. This is consistent with the “Anxiety Buffering Disruption Theory” [39]. Specifically, PTSD symptoms throw an individual's anxiety buffering system into a state of tension, or even collapse. This can lead to higher levels of anxiety. Therefore, to achieve better psychological intervention effects when managing anxiety symptoms among medical staff, attention should be paid to evaluating and managing possible PTSD symptoms.

This study's results showed that depression symptoms unidirectionally predicted subsequent anxiety symptoms, which is consistent with the previous studies [40, 41]. However, some scholars believed that a bidirectional predictive relationship exists between anxiety and depression symptoms [18]. Previous research found that in the general population, depression showed unidirectional causal dominance over anxiety, whereas in internet‐based cognitive‐behavioral therapy samples, neither depression nor anxiety exhibited causal dominance [42]. Our sample consisted of general medical staff (not in a treatment setting), which may explain why we observed a unidirectional pattern from depression to anxiety. Additionally, methodological choices such as symptom‐level versus disorder‐level analyses can affect results. Jacobson and Newman demonstrated that at the disorder level, depressive disorders strongly predicted certain anxiety disorders, whereas at the symptom level, the two conditions showed roughly equal bidirectional effects [18]. Considering that the relationship between anxiety and depression symptoms may change over time, and given the heterogeneity of findings across different populations and study designs, future research should further examine the cross‐time relationship between anxiety and depression symptoms by establishing surveys with three or more time points, and should also apply advanced causal inference methods to better clarify the directionality between them.

### 4.1 Limitations

Although we adopted a longitudinal study design to explore the dynamic change and the bidirectional predictive relationships of anxiety, depression, and PTSD symptoms, the current data only included two waves; therefore, the longitudinal predictive relationship should be interpreted cautiously. Future studies should conduct long‐term follow‐up surveys including three or more time points to further explore the predictive relationships between the study variables. Moreover, the sample was drawn from hospitals in Hubei Province, which may limit the generalizability of the findings. Finally, the small number of physicians and males limited the extrapolation of the research results, and in the future, emphasis should be placed on conducting multicenter, large‐sample surveys to improve the extrapolation of research results.

### 4.2 Clinical Implications

First, healthcare managers should recognize that major personal traumatic events predict elevated anxiety, depression, and PTSD symptoms among medical staff. A feasible management strategy is to encourage staff to voluntarily report such events and seek timely support. Managers can help by recognizing the impact of personal trauma on staff and offering simple support, such as brief counseling or flexible leave arrangements, when needed. Second, hospitals should implement targeted support for staff in high‐exposure roles, for example, regular group psychological debriefing and a confidential reporting system for workplace violence incidents. These measures can help mitigate the cumulative burden of occupational stress. Third, the asymmetric predictive relationships found in this study provide a basis for prioritizing specific intervention targets. The path from PTSD to depression was significantly stronger than the reverse path, suggesting that early screening for PTSD symptoms and timely trauma‐focused intervention may help prevent subsequent depression among medical staff. This does not mean ignoring depression, but rather recognizing that targeting PTSD early might yield additional benefits for depressive outcomes. Additionally, because PTSD and depression predicted subsequent anxiety, managers should be aware that medical staff presenting with PTSD or depression symptoms are at risk of developing anxiety later. When managing healthcare workers with PTSD or depression, monitoring for emerging anxiety symptoms is recommended.

## 5 Conclusions

The prevalence of anxiety, depression, and PTSD among medical staff was higher and showed an upward trend. This study confirmed a bidirectional predictive relationship between PTSD and depression, whereas PTSD/depression only unidirectionally predicted subsequent anxiety. Early screening and intervention for medical staff's anxiety, depression, and PTSD symptoms should be strengthened, and attention must be paid to the comprehensive management and intervention for anxiety, depression, and PTSD symptoms.

## Author Contributions

**Zongju Chen:** investigation, data curation, formal analysis, visualization, writing – original draft, writing – review and editing. **Ruiting Cai:** investigation, data curation, visualization, writing – original draft. **Yifang Liu:** conceptualization, investigation, data curation, writing – review and editing. **Na Tao:** methodology, writing – review and editing. **Yang Fei:** investigation, data curation, writing – original draft. **Jing Wen:** investigation, data curation, writing – original draft. **Pu Zhang:** investigation, writing – original draft. **Yu Lu:** investigation, writing – original draft. **Jun Zhang:** investigation, writing – original draft. **Jing Wang:** investigation, writing – original draft. **Li Zou:** conceptualization, investigation, supervision, writing – review and editing. **Wenning Fu:** conceptualization, methodology, supervision, writing – review and editing.

## Ethics Statement

The present study adhered to the principles of the Declaration of Helsinki, as revised in 2013. This study was approved by the Research Ethics Committee of the Tongji Medical College, Huazhong University of Science and Technology, Wuhan, China (No. 2021‐IEC‐A006). The ethical approval from Tongji Medical College, Huazhong University of Science and Technology has been acknowledged by the other participating hospitals.

## Consent

All the participants were asked to read and sign an informed consent form before accessing the link to complete the questionnaire.

## Conflicts of Interest

The authors declare no conflicts of interest.

## Supporting information


**Table S1:** The demographic characteristics of the participants who completed and did not complete follow‐up (*n* = 2751).
**Table S2:** Correlations between all model variables.

## Data Availability

The data sets used and/or analyzed during the current study are available from the corresponding author (Wenning Fu) on reasonable request.
