# Supplementary material for: Longitudinal Study on Post‐Traumatic Stress Disorder, Anxiety, and Depression Among Medical Staff in China
Source: Health Care Sci. 2026 Sep 13;5(5):404–14. doi: 10.1002/hcs2.70101 (PMC13571159; doi:10.1002/hcs2.70101)
Supplement: Supplementary file 1 [file HCS2-5-404-s001.docx]

**Supplementary Materials**

Table S1 The demographic characteristics of the participants who completed and did not complete follow-up (*n* = 2751)

| **Variables** | **Group** | **Completed follow-up,**  ***n* (%)** | **Not completed follow-up,**  ***n* (%)** | ***χ*^2^** | ***p*** |
| --- | --- | --- | --- | --- | --- |
| Age (years) | | | | | |
|  | 20–30 | 890 (33.58) | 43 (42.57) | 3.569 | 0.168 |
|  | 31–40 | 1370 (51.70) | 46 (45.54) |  |  |
|  | > 40 | 390 (14.72) | 12 (11.88) |  |  |
| Sex | | | | | |
|  | Male | 444 (16.75) | 17 (16.83) | 0.001 | 0.984 |
|  | Female | 2206 (83.25) | 84 (83.17) |  |  |
| Department | | | | | |
|  | Emergency | 69 (2.60) | 2 (1.98) | 0.742 | 0.690 |
|  | Intensive care unit | 182 (6.87) | 5 (4.95) |  |  |
|  | Others | 2399 (90.53) | 94 (93.07) |  |  |
| Occupational exposure | | | | | |
|  | Yes | 348 (13.13) | 16 (15.84) | 0.622 | 0.430 |
|  | No | 2302 (86.87) | 85 (84.16) |  |  |
| Exercise | | | | | |
|  | Always | 613 (23.13) | 29 (28.71) | 1.694 | 0.193 |
|  | Little or no | 2037 (76.87) | 72 (71.29) |  |  |
| Sleep quality | | | | | |
|  | Good | 623 (23.51) | 28 (27.72) | 1.582 | 0.663 |
|  | General | 1623 (61.23) | 56 (55.45) |  |  |
|  | Poor | 346 (13.06) | 14 (13.86) |  |  |
|  | Very poor | 58 (2.19) | 3 (2.97) |  |  |
| Experiencing traumatic life events | | | | | |
|  | Yes | 363 (13.70) | 8 (7.92) | 2.783 | 0.095 |
|  | No | 2287 (86.30) | 93 (92.08) |  |  |

Table S2 Correlations between all model variables

| **Variables** | **1** | **2** | **3** | **4** | **5** | **6** |
| --- | --- | --- | --- | --- | --- | --- |
| T1-PTSD | 1 | — | — | — | — | — |
| T1-Anxiety | 0.832^a^ | 1 | — | — | — | — |
| T1-Depression | 0.638^a^ | 0.638^a^ | 1 | — | — | — |
| T2-PTSD | 0.499^a^ | 0.436^a^ | 0.372^a^ | 1 | — | — |
| T2-Anxiety | 0.470^a^ | 0.448^a^ | 0.363^a^ | 0.831^a^ | 1 | — |
| T2-Depression | 0.383^a^ | 0.348^a^ | 0.383^a^ | 0.689^a^ | 0.707^a^ | 1 |
| *Note*: ^a^*p* < 0.01.  Abbreviations: PTSD, post-traumatic stress disorder; T1, the first phase of the survey; T2, the second phase of the survey; —, not applicable. | | | | | | |
